# Dynamic changes in ^18^F-borono-L-phenylalanine uptake in unresectable, advanced, or recurrent squamous cell carcinoma of the head and neck and malignant melanoma during boron neutron capture therapy patient selection

**DOI:** 10.1186/s13014-017-0949-y

**Published:** 2018-01-11

**Authors:** Takahiro Morita, Hiroaki Kurihara, Kenta Hiroi, Natsuki Honda, Hiroshi Igaki, Jun Hatazawa, Yasuaki Arai, Jun Itami

**Affiliations:** 1 Department of Diagnostic Radiology, National Cancer Center Hospital, 5-1-1 Tsukiji, Chuo-ku, Tokyo, 104-0045 Japan; 2 Department of Radiation Oncology, National Cancer Center Hospital, Tokyo, Japan; 3 Department of Nuclear Medicine and Tracer Kinetics, Osaka University Graduate School of Medicine, Osaka, Japan

**Keywords:** Dynamic ^18^F–BPA-PET study, Boron neutron capture therapy, Squamous cell carcinoma of the head and neck, Malignant melanoma

## Abstract

**Background:**

We evaluated dynamic changes in ^18^F–borono-L-phenylalanine (^18^F–BPA) uptake in unresectable, advanced, or recurrent squamous cell carcinoma of the head and neck (SCC) and malignant melanoma (MM) during boron neutron capture therapy (BNCT) patient selection.

**Methods:**

Dynamic changes in the maximum standardized uptake value (SUVmax), tumor-to-normal tissue ratio (TNR), and tumor-to-blood pool ratio (TBR) for ^18^F–BPA were evaluated in 20 patients with SCC and 8 patients with MM.

**Results:**

SUVmax in SCC tumors decreased significantly from 30 to 120 min. There was a non-statistically significant decrease in SUVmax for SCC tumors from 30 to 60 min and from 60 to 120 min. Patients with MM had nonsignificant SUVmax changes in ^18^F–BPA uptake on delayed imaging. Nonsignificant ^18^F–BPA TNR and TBR changes were seen in patients with SCC and MM.

**Conclusions:**

Dynamic changes in SUVmax for ^18^F–BPA uptake had a washout pattern in SCC and a persistent pattern in MM. Dynamic ^18^F–BPA -PET studies should be performed to investigate the pharmacokinetics of ^18^F–BPA in humans and select appropriate candidates who may benefit from BNCT.

## Background

Boron neutron capture therapy (BNCT) has been used for various types of intractable cancers, including glioblastoma, head and neck tumors, and melanoma [1–6]. This type of radiation therapy is based on nuclear reactions between neutrons and boron-10 (^10^B). After a targeted tumor contains a considerable concentration of ^10^B, the region to be treated is exposed to thermal neutrons. The nuclear reactions between these neutrons and ^10^B produce alpha particles and ^7^Li in a very short range (<10 μm) that should kill the cell. The success of BNCT depends on sufficient accumulation of ^10^B in tumor cells relative to adjacent tissues [5, 6]. Therefore, it is necessary to assess ^10^B concentration in tumor tissue before BNCT is performed [7].

Positron emission tomography (PET) using ^18^F–borono-L-phenylalanine (^18^F–BPA) has been used to screen for appropriate candidates who can benefit from BNCT [2, 3, 8–11]. Before BNCT, the ^10^B concentration in tumor tissue is estimated by measuring the tumor-to-normal tissue ratio (TNR) and the tumor-to-blood pool ratio (TBR) with ^18^F–BPA PET imaging [2, 3, 12, 13]. Hanaoka et al. demonstrated a significant positive correlation between levels of BPA and ^18^F–BPA accumulation in an animal model [14]. ^10^B accumulation is not consistent across patients; it is reported to also depend on tumor type [15, 16]. Thus, knowledge of the dynamic changes in ^10^B accumulation by tumor type is critical for performing BNCT. However, there is still limited information in the literature regarding dynamic changes in ^18^F–BPA uptake in various tumor types in humans. The purpose of this study was to examine the dynamic changes in the maximum standardized uptake value (SUVmax) of ^18^F–BPA in squamous cell carcinoma of the head and neck (SCC) and malignant melanoma (MM). TNR and TBR of ^18^F–BPA in SCC and MM were also evaluated.

## Methods

### General

The study protocol was approved by the institutional review board and independent ethics committee of our hospital. All patients provided written informed consent before inclusion in the trial.

### Radiosynthesis of ^18^F–BPA

^18^F–BPA was synthesized with direct electrophilic radiofluorination of BPA (Sigma-Aldrich, St. Louis, MO, USA) using ^18^F–acetyl hypofluorite as described previously [7, 17]. Purification of ^18^F–BPA was performed by high performance liquid chromatography (HPLC) using a YMC-Pack ODS-A column (20 × 150 mm; YMC, Kyoto, Japan) eluted with 0.1% acetic acid at a flow rate of 10 mL/min. The radiochemical purity of ^18^F–BPA as determined by HPLC was >99.5%. Its specific activity was 25 MBq/μmol.

### Patients and PET/CT protocol

This study included 20 patients with SCC and 8 patients with MM who underwent ^18^F–BPA PET/CT from March 2012 to August 2016. Patients had histologically confirmed malignant tumors and an Eastern Cooperative Oncology Group performance status of 0–1. We defined adequate organ function for patients with unresectable cancer on the basis of the normal range observed by our hospital laboratory. Adequate organ function was determined by neutrophil count ≥1500 /μL, platelet count ≥75,000 /μL, hemoglobin ≥9.0 g/dL, serum bilirubin ≤1.5 mg/dL, aspartate transaminase (AST) ≤ 100 IU/L, alanine aminotransferase (ALT) ≤ 100 IU/L, serum creatinine ≤1.5 mg/dL, and baseline left ventricular ejection fraction >60%. The main exclusion criteria were congestive heart failure, uncontrolled angina pectoris, arrhythmia, symptomatic infectious disease, severe bleeding, pulmonary fibrosis, obstructive bowel disease or severe diarrhea, and symptomatic pleural or pericardial effusion. This study was approved by the ethics committees of our institution.

Dynamic changes in ^18^F–BPA uptake were evaluated in 20 patients with SCC and 8 patients with MM. PET images were acquired using a Discovery 600 scanner (GE Healthcare, Milwaukee, WI, USA). PET images were reconstructed as using a 3D ordered-subset expectation maximization algorithm. PET image evaluation and quantification of SUV were performed using AW Volume Share 4.5 software. SUV was defined as regional radioactivity divided by injected radioactivity normalized to body weight. PET/CT images were taken 30, 60, and 120 min after ^18^F–BPA injection (4.0 MBq/kg of body weight). Regions of interest (ROIs) were drawn on the reconstructed PET images. Tumor SUVmax in ROIs was defined as the area of highest activity. ROIs were also drawn around normal tissue surrounding the tumor to calculate the TNR for ^18^F–BPA and the blood pool in order to calculate the TBR for ^18^F–BPA. The retention index (RI) was defined as the difference in SUVmax between early and delayed ^18^F–BPA PET imaging, expressed as a percentage of the initial uptake (RI = (SUVdelayed − SUVearly)/SUVearly × 100%). The difference in SUVmax and RI were calculated to evaluate the change in tracer levels in malignant lesions at 30, 60 and 120 min after ^18^F–BPA injection. Quantitative values above zero were defined as increased SUVmax and values below zero were defined as decreased SUVmax.

### Statistical analysis

SUVmax, TNR, and TBR were analyzed using paired one-way ANOVA. The paired t-test was used to determine the significance of differences in dynamic SUVmax values, TNR, and TBR. *P* < 0.05 was considered to indicate a statistically significant difference. For statistical analysis, JMP software (version 11.0, SAS Institute, Inc., Cary, NC, USA) was used.

## Results

Patient characteristics are summarized in Table 1. SUVmax, TNR, and TBR for ^18^F–BPA in SCC and MM are summarized in Table 2. Only SUVmax showed significant differences between 30 and 120 min in patients with SCC.

**Table 1:** Patient characteristics

Histology of the primary tumor	Number	Gender	Age, years
		Male/Female	Mean ± SD (range)
Squamous cell carcinoma	20	3/17	57.6 ± 16.1 (16–81)
Malignant melanoma	8	2/6	59.1 ± 13.7 (37–76)

*Abbreviation: SD* Standard deviation

**Table 2:** PET values for squamous cell carcinoma and malignant melanoma

PET value	Histology	^18^F–BPA at 30 min	^18^F–BPA at 60 min	^18^F–BPA at 120 min
		Mean ± SD	Mean ± SD	Mean ± SD
SUVmax	Squamous cell carcinoma	5.58 ± 2.29	4.79 ± 1.95	3.83 ± 1.56
	Malignant melanoma	9.41 ± 5.44	8.30 ± 4.61	7.39 ± 4.40
TNR	Squamous cell carcinoma	3.21 ± 1.66	3.28 ± 1.63	2.79 ± 1.52
	Malignant melanoma	7.89 ± 6.50	7.69 ± 4.91	6.68 ± 4.24
TBR	Squamous cell carcinoma	3.97 ± 1.76	3.84 ± 1.67	3.37 ± 1.52
	Malignant melanoma	9.82 ± 7.65	8.43 ± 4.33	8.33 ± 4.13

*Abbreviations:* ^*18*^*F–BPA* ^18^F–borono-L-phenylalanine, *PET* Positron emission tomography, *SD* Standard deviation, *SUVmax* Maximum standardized uptake value, *TNR* Tumor-to–normal tissue accumulation ratio, *TBR* Tumor-to-blood pool ratio

**Fig. 1:**
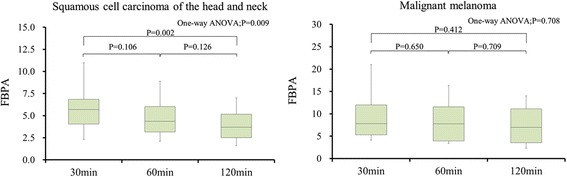
Box plot of SUVmax for tumors at 30, 60, and 120 min after ^18^F–BPA injection

Figure 1 is a box plot of SUVmax for tumors at 30, 60, and 120 min after injection. SUVmax in SCC tumors decreased significantly from 30 to 120 min, but the decrease was not statistically significant from 30 to 60 min and from 60 to 120 min. All 20 patients with SCC had gradual decreases in SUVmax from 30 to 120 min (Table 2). On the other hand. Nonsignificant ^18^F–BPA differences on delayed imaging were seen in patients with MM (Fig. 1, Tables 2 and 3). In contrast to patients with SCC, not all patients with MM had decreases in SUVmax from 30 to 60 min, 60 to 120 min, and 30 to 120 min.

**Table 3:** Statistically significant differences in dynamic SUVmax changes in squamous cell carcinoma and malignant melanoma

		Squamous cell carcinoma	Malignant melanoma
From 30 min to 60 min			
*P* value		0.1064	0.6504
Difference in SUVmax^a^	Mean ± SD (Range)	1.02 ± 0.61 (0.2–2.7)	1.1 ± 1.7 (−1.4–2)
RI	Mean ± SD (Range)	17.6 ± 7.3 (4.7–28.0)	12.0 ± 15.5 (−17.1–29.9)
	Number of patients with increase	0	3
	Number of patients with decrease	20	5
From 60 min to 120 min			
*P* value		0.1263	0.7098
Difference in SUVmax	Mean ± SD (Range)	0.96 ± 0.56 (0.3–2.6)	0.91 ± 0.74 (−0.2–2.3)
RI	Mean ± SD (Range)	20 ± 6.6 (5.2–29.2)	13.6 ± 10.9 (−0.02–32.4)
	Number of patients with increase	0	1
	Number of patients with decrease	20	7
From 30 min to 120 min			
*P* value		0.0023	0.412
Difference in SUVmax	Mean ± SD (Range)	1.98 ± 1.02 (0.6–4.7)	2.03 ± 2.38 (−1.1–7)
RI	Mean ± SD (Range)	34.0 ± 8.7 (8.7–46.7)	22.4 ± 22.3 (−13.4–52.1)
	Number of patients with increase	0	2
	Number of patients with decrease	20	6

^a^Difference in SUVmax difference was calculated as delayed SUVmax minus earlier SUVmax

*Abbreviations: RI* Retention index, *SD* Standard deviation, *SUVmax* Maximum standardized uptake value

**Fig. 2:**
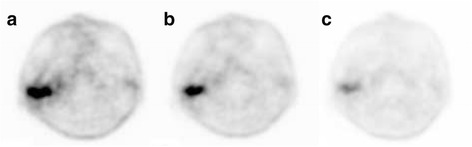
Representative ^18^F–BPA PET images in a 50-year-old man with squamous cell carcinoma of the external auditory canal. ^18^F–BPA PET images at (**a**) 30 min (SUVmax = 11.0, TNR = 5.0, TBR = 8.5), (**b**) 60 min (SUVmax = 8.9, TNR = 5.2, TBR = 6.9), and (**c**) 120 min (SUVmax = 6.3, TNR = 4.5, TBR = 5.3) after injection

**Fig. 3:**
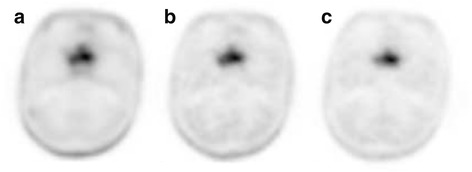
Representative ^18^F–BPA PET images of malignant melanoma in a 39-year-old woman with sphenoid sinus melanoma. ^18^F–BPA PET images at (**a**) 30 min (SUVmax = 8.2, TNR = 7.5, TBR = 5.9), (**b**) 60 min (SUVmax = 9.6, TNR = 9.6, TBR = 6.9), and (**c**) 120 min (SUVmax = 9.3, TNR = 9.3, TBR = 7.2) after injection

Nonsignificant TNR and TBR for ^18^F–BPA were seen on delayed imaging in both patient groups (Table 2). Representative ^18^F–BPA PET images are shown in Figs. 2 and 3.

## Discussion

The aim of this study was to examine dynamic ^18^F–BPA changes in SUVmax in SCC and MM as part of the patient selection process for BNCT. In SCC, dynamic changes in SUVmax for ^18^F–BPA uptake had a washout pattern, compared with a persistent pattern of ^18^F–BPA uptake in MM.

^18^F–BPA was developed to predict ^10^B accumulation in tumors and normal tissues with PET [18]. Studies have shown that there are a variety of amino acid transporters, such as Systems L, A, ASC, and B [19, 20]. System L is the primary contributor to ^18^F–BPA uptake, which is correlated with total L-amino acid transporter (LAT) expression, more specifically LAT1 and LAT4. Many tumors overexpress LAT1 or LAT4 [21–23]. Previous studies have shown that the expression of amino acid transporters in tumors varies widely, and it sometimes reflects proliferation speed and malignancy [24]. However, reasons for differences in dynamic changes in ^18^F–BPA uptake between SCC and MM remain uncertain. It is unclear whether ^18^F–BPA undergoes metabolic transformation, although metabolic transformation of L-phenylalanine has been reported [25]. LAT and the metabolic transformation of ^18^F–BPA may contribute to dynamic changes in ^18^F–BPA accumulation in tumors. Further studies with more participants and evaluation of processes involved in ^18^F–BPA metabolic transformation are needed to resolve this question.

In clinical BNCT, ^18^F–BPA accumulation was measured about 1 h after ^18^F–BPA administration [26–29]. However, the number of dynamic studies of ^18^F–BPA uptake in humans is limited. Therefore, we focused on dynamic ^18^F–BPA uptake in humans. Our study showed that SUVmax for ^18^F–BPA uptake in SCC has a washout pattern. It is very important to realize that some tumor histological types may have a washout pattern. ^18^F–BPA uptake in different tumor types may be vary with extended distribution time in ^18^F–BPA PET imaging. Further dynamic ^18^F–BPA -PET studies should be performed to determine who are appropriate candidates that can benefit from BNCT.

In this study, we did not evaluate the pharmacokinetics of BPA or the BPA-fructose complex because we focused on dynamic accumulation of ^18^F–BPA in human tumors. Hanaoka et al. showed a positive association between the levels of BPA and ^18^F–BPA accumulation in a rat model [14]. However, the biodistribution of ^18^F–BPA in animals and humans is different [30]. In addition, metabolic transformation of ^18^F–BPA and BPA in vivo may also differ. Direct pharmacokinetic comparisons between ^18^F–BPA and BPA levels in tumors are required during and at the end of BNCT in humans to define early and delayed ^18^F–BPA imaging times.

The present study had some limitations. Two different tumor types were examined in our study. Our ^18^F–BPA findings for SCC were consistent with previous studies [28, 31]. However, the characteristics of dynamic ^18^F–BPA accumulation in radioresistant head and neck carcinomas, such as mucoepidermoid carcinomas and adenoid cystic carcinomas, is unknown [3]. Various intractable cancers that can be treated with BNCT represent a wide spectrum of histopathological backgrounds. Further studies involving more patients, each representing a specific pathological entity, are therefore needed.

## Conclusions

Dynamic changes in SUVmax for ^18^F–BPA uptake in SCC has a washout pattern, while ^18^F–BPA uptake in MM has a persistent pattern. Dynamic ^18^F–BPA -PET studies should be performed as part of a human pharmacokinetic study of ^18^F–BPA and to select appropriate candidates who may benefit from BNCT.

## Abbreviations

^18^F–BPA: ^18^F–borono-L-phenylalanine;
ALT: Alanine aminotransferase
AST: Aspartate transaminase
BNCT: Boron neutron capture therapy
HPLC: High-performance liquid chromatography
LAT: System L amino acid transporter
MM: Malignant melanoma
PET: Positron emission tomography
RI: Retention index
ROIs: Regions of interest
SCC: Squamous cell carcinoma of the head and neck
SUVmax: Maximum standardized uptake value
TBR: Tumor-to-blood pool ratio
TNR: Tumor-to–normal tissue accumulation ratio
